# Selective Excitation of Exciton–Polariton Condensate Modes in an Annular Perovskite Microcavity

**DOI:** 10.1021/acs.nanolett.4c00634

**Published:** 2024-04-15

**Authors:** Zhenyu Xiong, Hao Wu, Yuanwen Cai, Xiaokun Zhai, Tong Liu, Baili Li, Tieling Song, Longfei Guo, Zhengliang Liu, Yifan Dong, Peicheng Liu, Yuan Ren

**Affiliations:** † Department of Aerospace Engineering and Technology, Space Engineering University, Beijing 101416, China; ‡ Lab of Quantum Detection & Awareness, Space Engineering University, Beijing 101416, China; § Institute of Molecular Plus, Tianjin University, Tianjin 300072, China

**Keywords:** exciton−polariton, bosonic condensation, selective excitation, perovskite microcavity, light field regulation

## Abstract

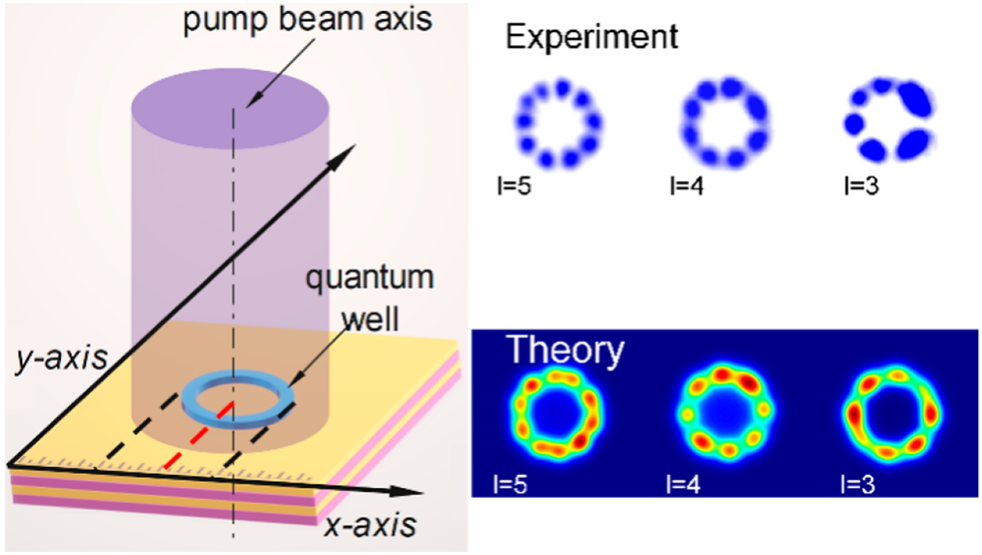

Exciton–polariton systems composed of a light–matter quasi-particle with a light effective mass easily realize Bose–Einstein condensation. In this work, we constructed an annular trap in a halide perovskite semiconductor microcavity and observed the spontaneous formation of symmetrical petal-shaped exciton–polariton condensation in the annular trap at room temperature. In our study, we found that the number of petals of the petal-shaped exciton–polariton condensates, which is decided by the orbital angular momentum, is dependent on the light intensity distribution. Therefore, the selective excitation of perovskite microcavity exciton–polariton condensates under all-optical control can be realized by adjusting the light intensity distribution. This could pave the way to room-temperature topological devices, optical cryptographical devices, and new quantum gyroscopes in the exciton–polariton system.

Exciton–polaritons, arising from the strong coupling of excitons and photons in semiconductor microcavities, exhibit unique properties as hybrid quasi-particles with characteristics of both light and matter. As early as the late 1950s, Hopfield put forward the concept of an exciton–polariton.^1^ Over the past several decades, significant progress has been made in the study of exciton–polaritons, particularly in the realm of microcavity research.^2−5^ Notably, the observation of the anticrossing dispersion behavior of polaritons has unveiled their potential for Bose–Einstein condensation (BEC).^6−9^ Microcavity exciton–polariton condensation has demonstrated a remarkably light effective mass, making it readily attainable.^9−13^ Moreover, the critical temperature for condensation can reach several kelvin, and advancements have been made toward achieving room-temperature condensation in materials such as GaN,^13−17^ organic compounds,^18−20^ and halide perovskite materials,^21−24^ surpassing other bosonic systems. In practical applications, microcavity exciton–polariton systems and their control mechanisms find utility in optical switches,^25^ cryptographic devices,^26^ and neural networks.^27,28^

Perovskite semiconductor materials, renowned for their versatility and ease of fabrication, have found extensive applications in the field of optics and optoelectronics.^29−33^ These materials offer a convenient platform for harnessing exciton–polaritons due to their facile preparation and ability to form optical microcavities. Via the introduction of engineered microstructures into samples, it becomes possible to manipulate the condensate modes of excitons within microcavities.^34,35^ Furthermore, compared to low-temperature materials, materials operating at room temperatures, such as those used in organic^36^ and perovskite^37,38^ materials, enable us to manipulate the electric field. This facilitates the study of exciton polarization and exciton abundance phenomena across multiple dimensions.

In this study, we focus on CsPbBr_3_ perovskite samples, in which a patterned thin film is deliberately incorporated into the microcavity as an upper layer using positive photoresist lithography. Through excitation with Gaussian femtosecond pulse lasers, we observe the emergence of room-temperature Bose–Einstein condensates within the annular trap, exhibiting a distinctive petal-shaped distribution in real space. By conducting interference measurements in real space, we ascertain a π phase difference between adjacent petals of the condensate. Notably, we observe petal-shaped exciton–polariton condensates with varying petal numbers within the same microcavity dimensions. Furthermore, our experimental findings reveal a regular variation in the number of petals by manipulating the light intensity distribution near the annular trap, consistently near the same energy level in k-space. This work elucidates the generation of higher-order modes of BEC at room temperature and provides insights into mode-selective excitation via tailored light field distributions in annular traps. Unlike previous reports,^39,40^ we achieve multimode switching of exciton–polaritons at room temperature by adjusting the pump light.

The perovskite microcavity structure adopted in this work is shown in Figure 1a, with a CsPbBr_3_ perovskite layer and annular positive photoresist layer sandwiched between two distributed Bragg reflectors (DBR). In the experiment, we used a variety of annular trap sizes with an inner ring diameters of 4, 8, and 10 μm, while the width of the rings is fixed at 2 μm.

**Figure 1:**
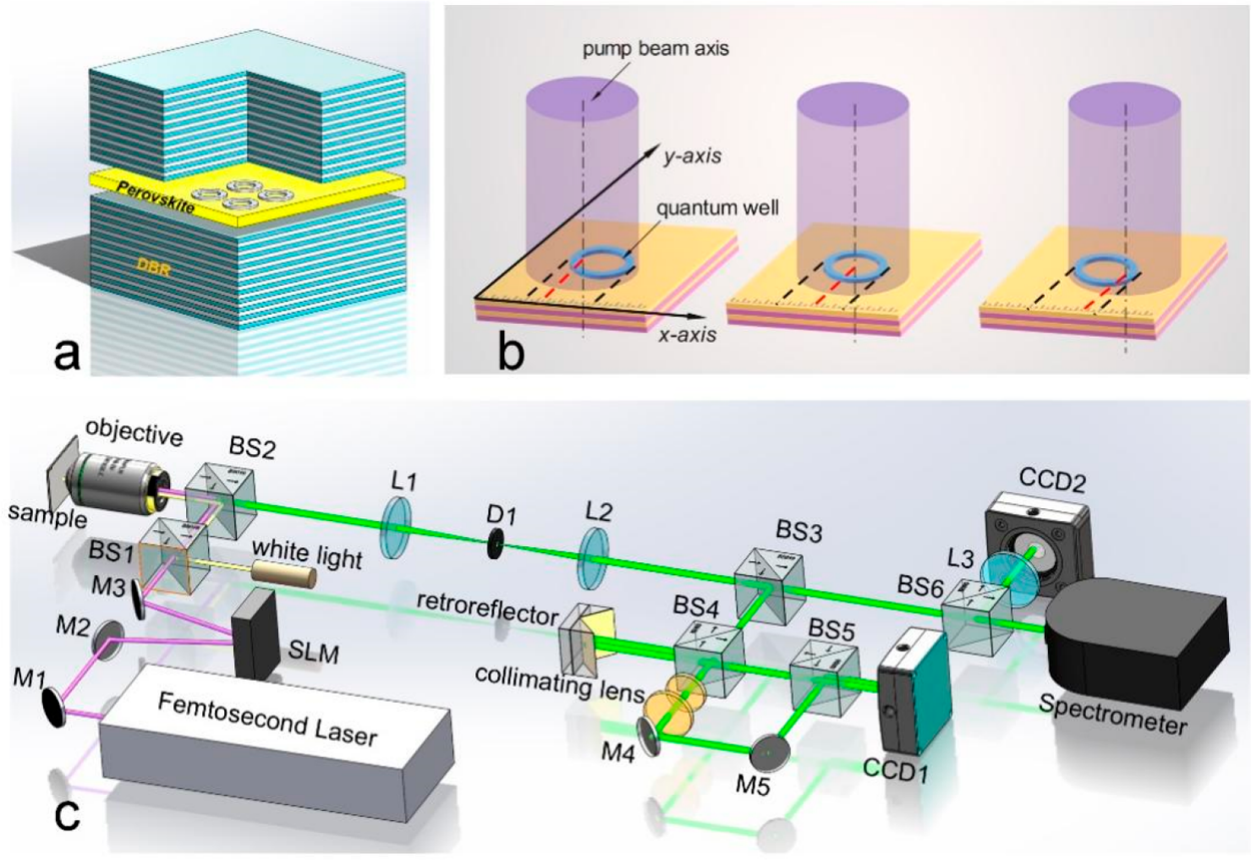
(a) Schematic diagram of the annular perovskite microcavity, which is composed of two distributed Bragg mirrors on the upper and lower sides. (b) Schematic diagram of light field control. SLM is used to control the relative displacement between the center of the spot and the annular trap. (c) Optical path diagram adopted in the experiment, in which the femtosecond pulse beam is modulated by SLM and the photoluminescence signals are sent to the interference loop, camera, and spectrometer.

The CsPbBr_3_ perovskite samples are excited by femtosecond pulse pumping in this work. The wavelength of the pump light is 400 nm, the pulse repetition frequency 6 kHz, with a 190 fs pulse width, and the pump light intensity a Gaussian type distribution. As shown in Figure 1c, pumped light is irradiated on the liquid crystal spatial light modulator (SLM) after beam expansion, and the diffracted light spot is irradiated on the surface of the sample through a microscopic objective with a numerical aperture of 0.8 forming a Gauss distribution spot with a diameter of ∼70 μm. By changing the hologram loaded on the SLM, we changed the diffraction angle of the diffracted light.

We first load the hologram onto the SLM, and the modulated light is focused on the sample plane through a microscopic objective after collimation (see section II of the Supporting Information for more details of the light path). The modulated Gaussian type pump light is used to pump the annular trap; the photoluminescence intensity of the sample in real space increases with the pump light power. When the power of the pump light exceeds the excitation threshold, the luminescence in the ring-shaped trap increases sharply. Meanwhile, the petal-shaped luminous regions and interference fringes are observed in real space, and their dispersion curves show the condensation process, as shown in Figure 2.

**Figure 2:**
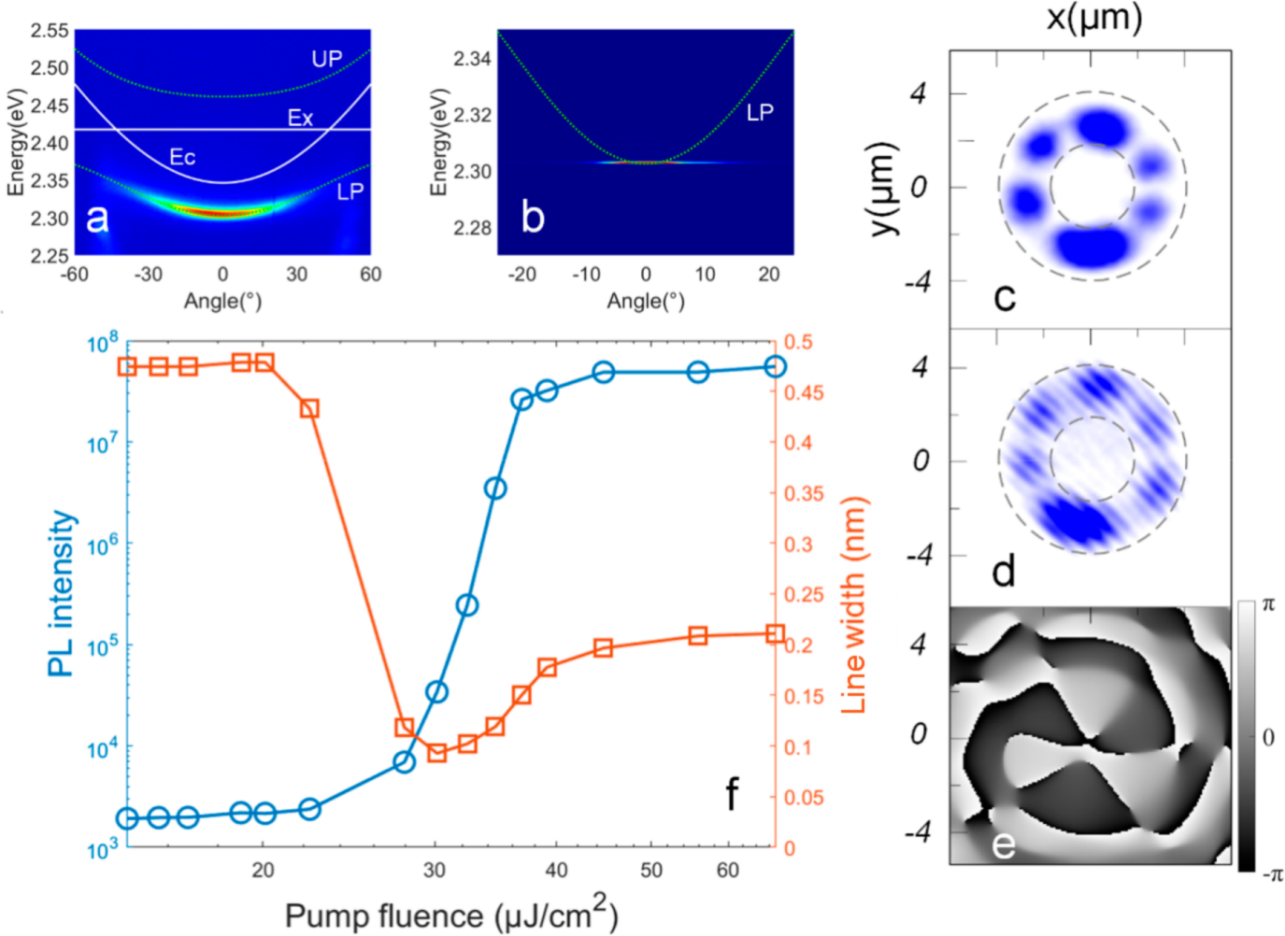
(a and b) Dispersion curves of the annular CsPbBr_3_ perovskite sample below and above, respectively, the threshold values measured in the experiment. The green dashed line shows the lower polariton branch that is fitted. It is noteworthy that the coordinate range in panel b is smaller than that in panel a to emphasize the relationship between the fitting curve of exciton polaritons above and below the threshold. (c and d) *l* = 3 mode exciton–polariton condensate measured in one experiment and the corresponding interference diagram, respectively. (e) Phase information that corresponds to panels c and d. (f) Evolution of the photoluminescence intensity (blue line) and line width (red line) with pump power density in the annular perovskite sample.

In the experiment, the petal-shaped exciton–polariton (EP) condensates always have an even number of petals. This petal-shaped mode of exciton–polariton condensation has been observed in previous experiments and theory,^41−43^ and it is a coherent orbital coupling of counter-rotating exciton–polaritons. To measure the phase information on the condensate, the petal-shaped exciton–polariton condensates are introduced into an improved Mach–Zehnder interferometer (see section IV of the Supporting Information). After passing through the interference loop, interference fringes appeared, indicating the presence of long-range coherence in the condensate. Additionally, we observe that the interference fringes are discontinuous and exhibit dislocation phenomena. As shown in Figure 2c–e, by calculating the phase through interference, we find that the phase distribution in the exciton–polariton condensate also follows a petal-shaped pattern with the same number of petals as the condensate. This suggests that each petal possesses a well-defined phase, with a phase difference of π between each petal and its two adjacent petals. In the context of vortex optics, these properties are consistent with the superposition of two Laguerre–Gauss (LG) modes with a topological charge of *±l*, where *n* = 2*l* represents the number of petals.^34^

Variation of petal-shaped exciton–polariton condensates in the same size ring-shaped trap has been achieved by changing the light intensity distribution with the SLM. For instance, three exciton–polariton condensate modes with *l* = 3, 4, and 5 can be found in the sample with an inner diameter of 4 μm. Figure 3 shows the real-space petal modes and corresponding k-space dispersion curves of the three modes; at the same time, we find that all three modes are in the same lower polariton branch (LPB).

**Figure 3:**
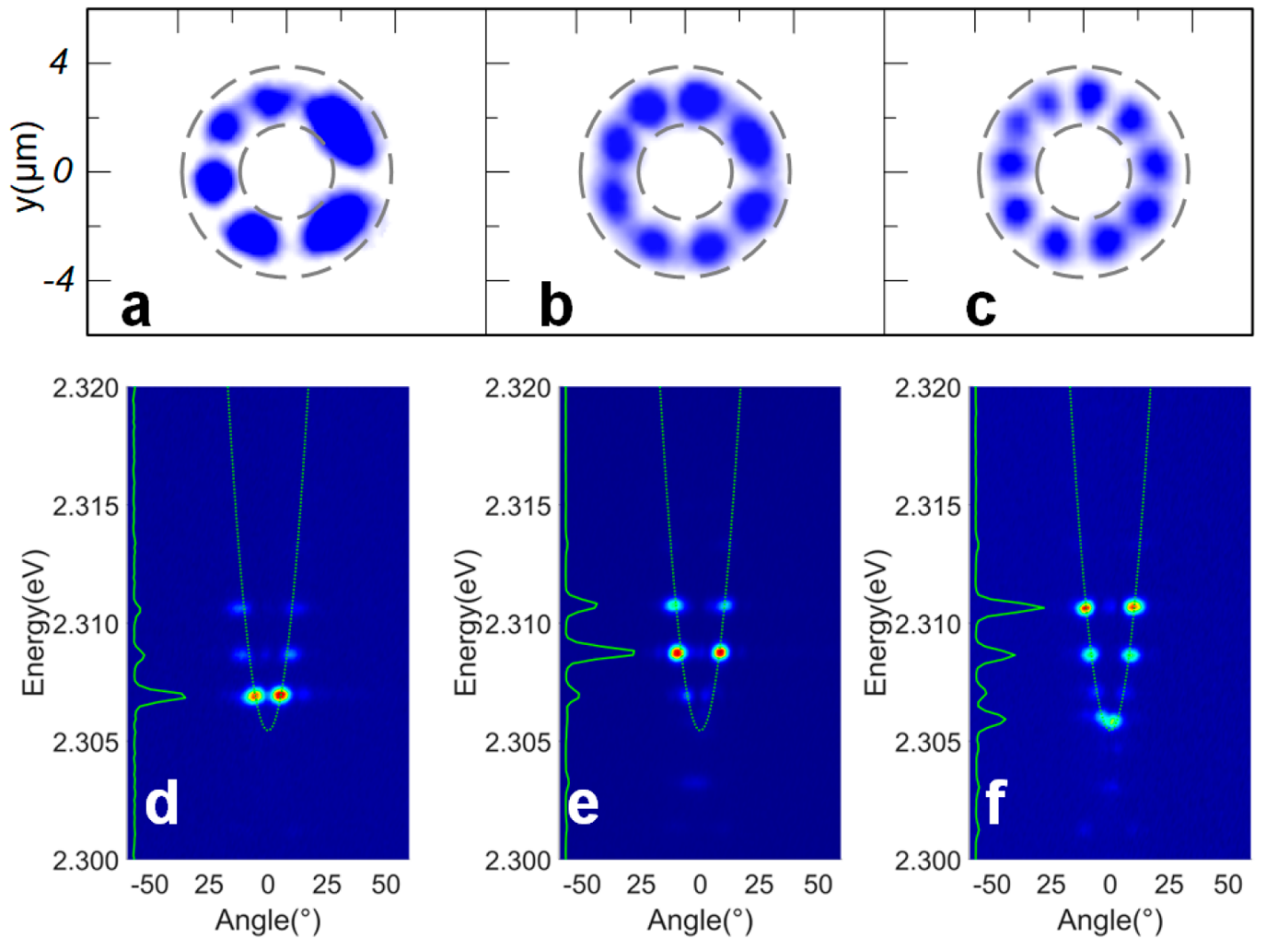
(a–c) Real-space modes of the three petal-shaped exciton–polariton condensates observed in the experiment, corresponding to the *l* = 3, 4, and 5 modes, respectively. The dotted gray line indicates the location of the ring-shaped trap. (d–f) Momentum-space diagram corresponding to panels a–c, respectively, of the three modes. The green solid line at the ordinate indicates the luminous spectral intensity. One can clearly see that there are three peaks, and each real-space mode corresponds to a peak in the dominant position.

Through analysis of the k-space spectrum, we have discovered that exciton–polaritons can condense in three distinct energy levels, each corresponding to three different modes. As shown in Figure 3, these three modes’ energy levels simultaneously exist in the k-space spectrum. However, due to their varying relative strengths, we can observe only the real-space distribution that corresponds to the dominant mode in physical space.

As shown in Figure 1c, we manipulate the diffraction angle of the diffracted light after SLM via the regulation of the hologram loaded on the SLM, and thus, the light spot of the Gaussian light pump focusing through the microscopic objective lens moves in two dimensions on the surface of the sample. In the process of spot movement, it is found that when the spot center (that is, the strongest point of the light intensity distribution) of the Gauss-pumped laser aligns with the center of the ring-shaped trap, the distribution of the petal-shaped exciton–polariton condensates generated in the annular trap shows a higher number of petals in real space and a higher energy level in the measurement of k-space is observed. When the spot center of the Gaussian-pumped laser deviates from the center of the annular trap, the distribution of the petal-shaped exciton–polariton condensates generated by excitation in the annular trap transforms into a smaller number of petals in real space with a lower energy level measured in k-space.

Furthermore, to obtain the detailed correlation between the three modes in real space and k-space evolution in, we generated a series of holograms to quantitatively vary the displacement of pump light at the sample plane after each hologram switch. To obtain better contrast, a simple one-dimensional linear displacement is adopted here, and the displacement amount is controlled at 2 μm each time. The initial position is denoted when the spot center of the Gaussian-pumped laser coincides with the center of the annular trap, and the displacement is denoted as 0 μm. On the basis of the initial position, the movement of the pump light position is controlled, and a series of data are measured and then plotted as shown in the figure (here we analyze only the *l* = 3, 4, and 5 modes, which appear above, ignoring other modes).

As shown in Figure 4, the laser intensity of the three modes is extracted and normalized while the intensity distribution of the light field is continuously being changed. When the intensity of the light field distributed in the annular trap is approximately symmetric, the dominant mode of the BEC in the microcavity is the *l* = 5 mode in a relatively high energy state. When the intensity of the light field distributed in the annular trap has a certain asymmetry, the microcavity BEC mode is dominated by the *l* = 4 mode in the intermediate energy state. Furthermore, the asymmetry of the intensity distribution of the light field is further increased; the real-space distribution of the *l* = 3 mode is found in the annular trap, and the corresponding energy of this mode is relatively low among the three. This correlation between energy levels indicates that the *l* = 3 mode is in a more stable state while the *l* = 5 mode is in an unstable state. The *l* = 3 mode with a lower energy can remain dominant in a relatively large and uneven distribution of light intensity, and the highest proportion of the highest mode is also the highest among the three. In contrast, the *l* = 5 mode is in a relatively high energy state among the three modes, and its mode is less stable and easy to convert into other modes. Therefore, the *l* = 5 mode is dominant only in a small range of adjustment in the light intensity distribution, and the maximum mode proportion that can be achieved is also the lowest among the three modes. At the same time, we noticed that in our experimental results, the switching of the three modes appeared on the same LPB, which was different from the switching of the modes that appeared on different LPBs before H.b Fu.^36^

**Figure 4:**
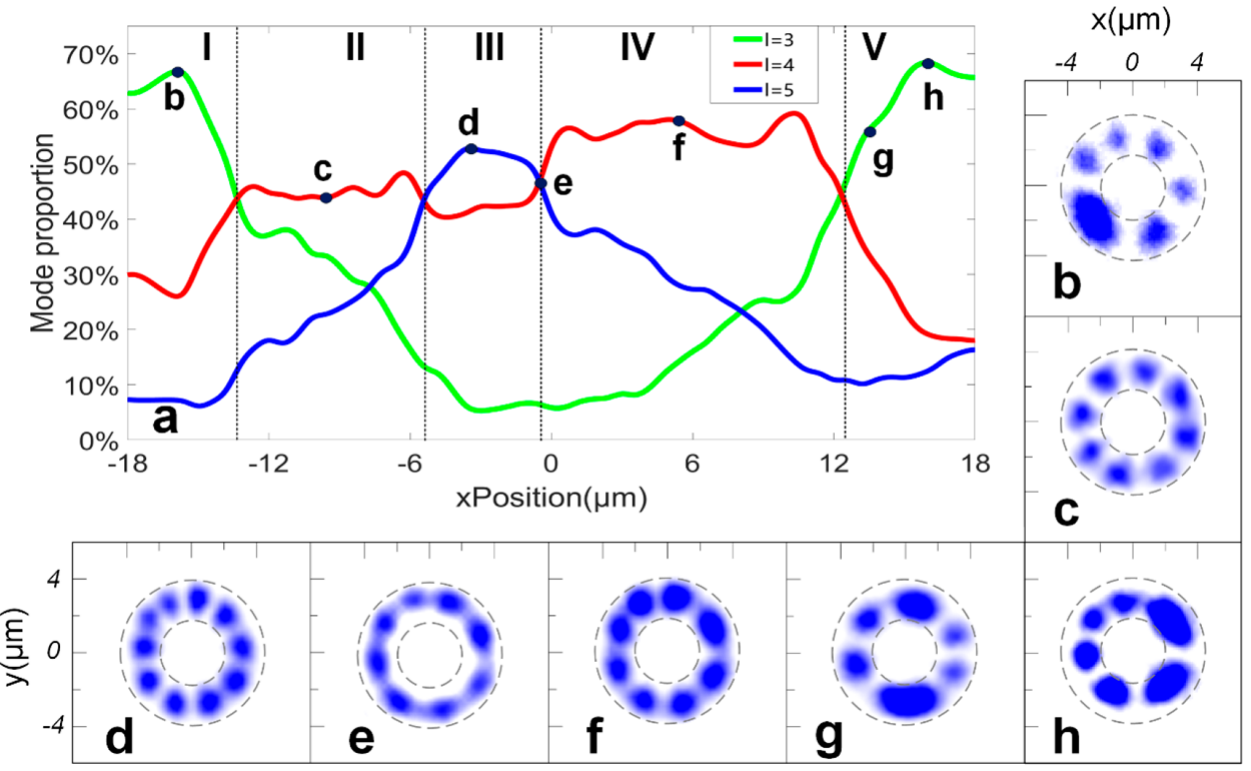
Relationship between the proportion of the three microcavity BEC modes and the displacement under one-dimensional motion. (a) Shift in the spot position in relation to the proportion of *l* = 3 (green), *l* = 4 (red), and *l* = 5 (blue) modes, where the weighted sum of the lasing intensity of the three modes is 100% plotted. (b–h) Real-space modes at each point in panel a. The dotted gray line indicates the location of the ring-shaped trap.

On the basis of the research findings presented above, we observed that when the pumping light intensity distribution is relatively uniform in the annular trap the exciton–polariton in the microcavity tends to condense at a higher energy level, forming multiple petals in the condensates. Conversely, if the pumping light intensity distribution is not uniform enough, the exciton–polariton condenses at a lower energy level, resulting in fewer petals in the condensate. This indicates that the non-uniform distribution of pumping light intensity affects the terms related to pumping in the Gross–Pitaevskii (GP) equation.^44^ We speculate that this is caused by the optical gradient arising from the non-uniform distribution of light intensity. In common pumping excitation systems, Gaussian pumping lasers are typically used and the pumping is adjusted to achieve a relatively uniform intensity distribution after beam expansion. Previous research teams have found that exciton–polaritons in the ring traps of ring microcavity systems spontaneously condense at different energy levels,^34^ while the energy levels are discretely distributed in the same annular trap. On the contrary, in the exciton–polariton condensate system, an increase in light intensity leads to a blue-shift. In our study, we deliberately controlled the non-uniform distribution of light intensity in the annular trap, resulting in variations in the pumping intensity in different regions. Therefore, the region with weaker pumping displayed a red-shift. Nevertheless, because of the interconnectedness of the annular trap, the exciton–polaritons in the system eventually collectively underwent a transition to lower energy levels and were stabilized in an alternative mode. Building upon the previously mentioned GP equation, we adjusted the pumping term and derived theoretical outcomes that corroborate the experimental observations (see section VII of the Supporting Information).

In short, we can selectively excite three different modes of exciton–polariton condensates in CsPbBr_3_ perovskite materials by controlling the intensity of the light field distribution in the annular trap. These three modes are located on the same LPB in k-space and exhibit different petal-shaped spatial distributions in real space. Each of the three petal-shaped polariton condensates corresponds to a different mode. In further studies, we found that the intensity distribution of the light field near the annular trap affects the mode of exciton–polariton condensation. We describe the influence of the intensity distribution of the light field on the three exciton–polariton modes and provide a method for manipulating the light field intensity to produce these three modes. The similar results obtained from theoretical simulations further confirm the manipulation of exciton modes by the light field distribution. Our experiment introduces a new feasible method for selectively exciting exciton–polariton condensation modes in annular perovskite microcavities using all-optical means, which offers a reference for realizing all-optical regulation of microcavity polaritons in the future.

## Supporting Information Available

Preparation and microscope photographs of CsPbBr_3_ perovskite samples, experimental light path diagrams and procedures, development and validation of theoretical models, construction of interference rings and phase diagrams of various petal-shaped modes, and our experimental results at different pump light sizes or in different annular traps (PDF)
