# Supplementary material for: Selective Excitation of Exciton–Polariton Condensate Modes in an Annular Perovskite Microcavity
Source: Nano Lett. 2024 Apr 15;24(16):4959–64. doi: 10.1021/acs.nanolett.4c00634 (PMC11057030; doi:10.1021/acs.nanolett.4c00634)
Supplement: Supplementary file 1 — nl4c00634_si_001.pdf [file nl4c00634_si_001.pdf]

SUPPLEMENTARY MATERIAL for

Selective excitation of exciton-polariton condensate modes in annular perovskite microcavity

Zhenyu Xiong[1,#],Hao Wu[1,2,#],Yuanwen Cai[1],Xiaokun Zhai[3],Tong Liu[1],Baili Li[2],Tieling Song[2],Longfei Guo[2],Zhengliang Liu[1],Yifan Dong[1],Peicheng Liu[2],Yuan Ren[1,2,*]

1, Department of Aerospace Engineering and Technology, Space Engineering University
2, Lab of Quantum Detection & Awareness, Space Engineering University
3, Institute of Molecular Plus, Tianjin University

#: These authors contributed equally to this work
*: Corresponding author

Corresponding Author
*Yuan Ren, Lab of Quantum Detection & Awareness, Space Engineering University
Email : renyuan_823@aliyun.com

Supplemental information I. Preparation and characterization of experimental materials

The structure of the perovskite microcavity includes a bottom distributed Bragg reflector (DBR), a $CsPbBr_3$ perovskite layer, a positive photoresist layer, and a top DBR layer. To obtain a larger perovskite sample, the $CsPbBr_3$ perovskite is initially grown on mica, resulting in a square perovskite sample with thickness of 100-200 nm. Subsequently, the perovskite on the mica is transferred onto the bottom DBR. A 120 nm positive photoresist is then spin-coated onto another DBR and shaped into a annular form using lithography to create a annular trap for capturing the

exciton polariton. Finally, the two DBRs are pressed together to form the final experimental material. In order to eliminate the mutual coupling between different annular traps, the interval between the center of adjacent annular traps is set to be 50 μm when designing annular traps.

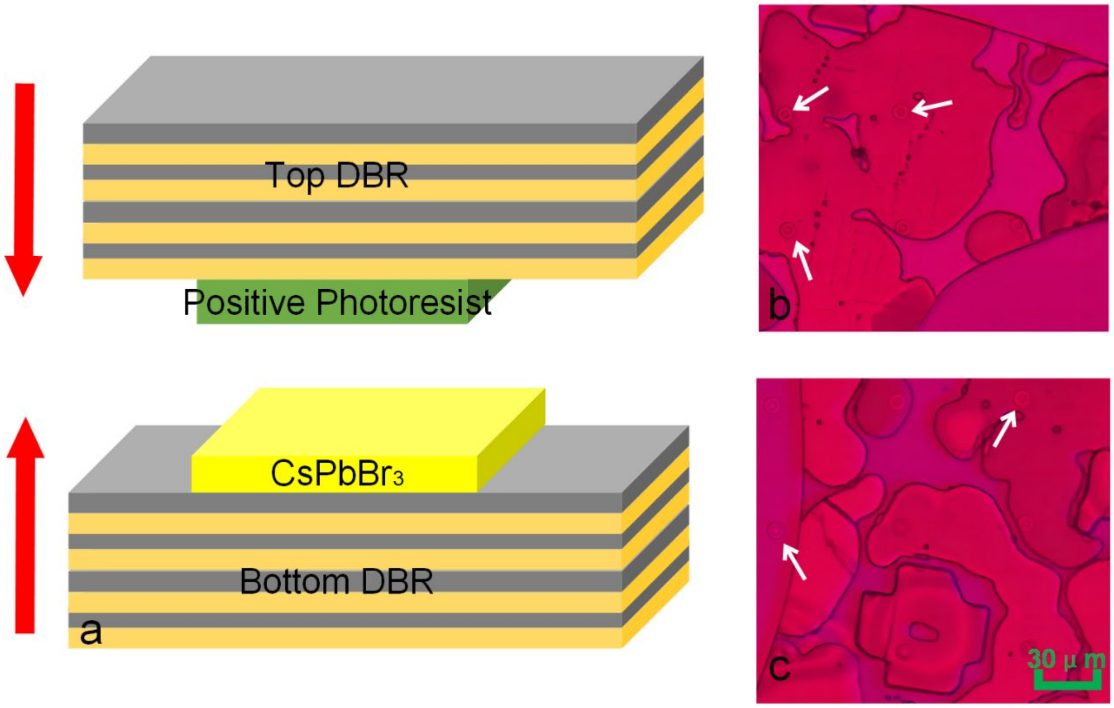

FIG. S1 (a) Material structure and preparation process, (b) and (c) microscope images of samples with annular microcavity sizes of 4-8 μm, and the rings are about 50 μm apart. The scale in the figure represents 30 μm.

It is important to note that when $CsPbBr_3$ perovskite is grown on the mica substrate, covalent bonds are formed between the mica and perovskite. As a result, during the transfer process, a thin layer of mica may adhere to the perovskite, creating a very thin mica layer between the DBR and perovskite at the bottom. However, for the purposes of the experiment, this thin mica layer can generally be disregarded.

Supplemental information II. Optical pump and measuring device

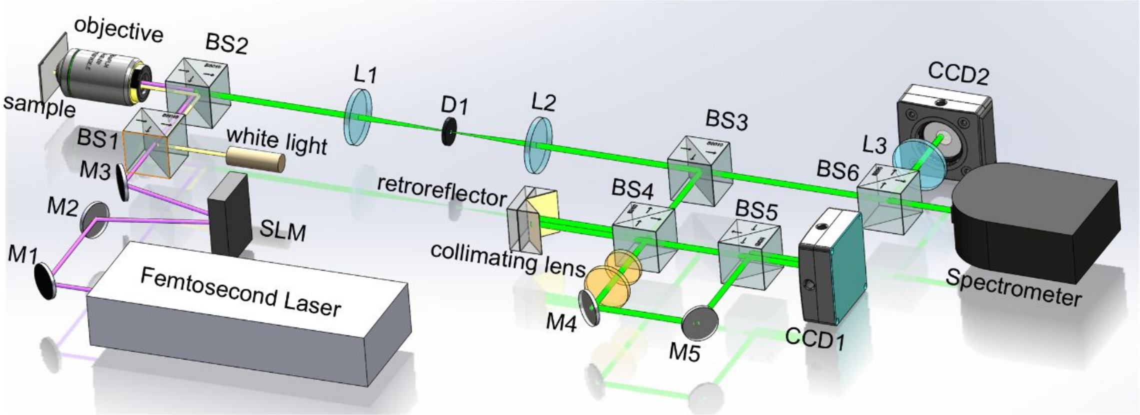

FIG. S2: Experimental light path diagram

The figure above shows the optical path used in the experiment. The femto-second laser emits a pump light with a wavelength of 400 nm, a pulse repetition rate of 6 KHz, a pulse width of 190 fs, and a Gaussian intensity distribution. After beam expansion, the pump light irradiates the liquid crystal spatial light modulator (SLM), and the diffraction spot irradiates the sample surface through the microscope objective with a numerical aperture of 0.8, forming a Gaussian spot with a diameter of ~70 μm. By changing the hologram loaded on the SLM, the diffraction angle of the diffracted light can be slightly altered, and then the spot can be controlled to move slightly in the focal plane of the microscope objective (the sample surface). The 4f system collects the fluorescence signal and divides it into three channels. One channel detects the fluorescence signal in real space by camera, another channel sends the signal to the improved Mach-Zehnder interferometer (see Supplemental information IV) to explore the coherence characteristics of the

fluorescence signal, and the last channel realizes the spectrometer in k-space by spectrometer.

Supplemental information III. Exciton polariton blue shift and real space PL images

Graph of the relationship between the pump fluence measured at the annular trap and the PL intensity, linewidth and blue shift.

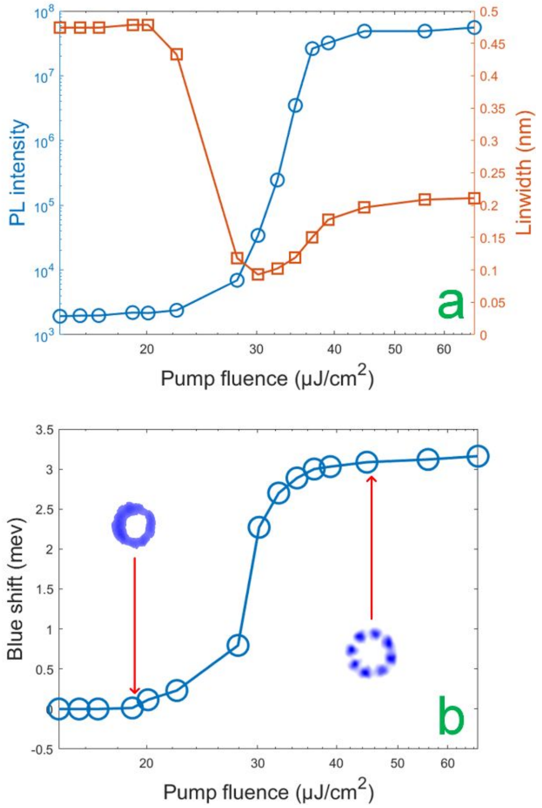

FIG.S3 The relationship between the pump fluence measured at the annular trap and the PL intensity, linewidth and blue shift. (a)The evolution of the photoluminescence intensity (blue line) and line width (red line) with the pump power density in the annular perovskite sample. (b)The evolution of the photoluminescence intensity with the pump power density in the same annular perovskite sample. The two small figures correspond to the real-space emission patterns below and above the threshold respectively.

Supplemental information IV. Construction and result of interference light loop

We utilize an improved Mach-Zehnder interference loop to achieve the long-range coherence of exciton-polariton condensates[1, 2], as depicted in FIG.S4. The luminescence of the condensates is divided into two beams using a beam splitter mirror. One beam is reflected, while the other beam is expanded through a pair of aligned lenses. By adjusting the optical path difference, the two fluorescence signals are temporally aligned, resulting in the observation of interference fringes. This observation verifies the long-range coherence of exciton-polariton condensates.

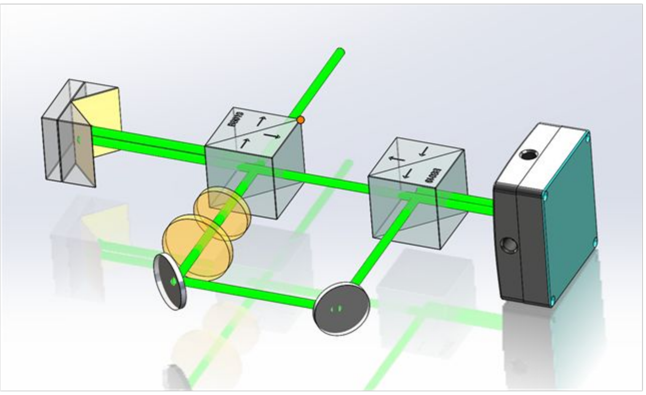

FIG.S4: Schematic diagram of an improved Mach-Zehnder interference loop, in which the expanded beam interferes with the original beam after passing through an aligned straight lens.

In addition, under pumping excitation, exciton-polariton condensates in the shape of petals are generated. These petal-shaped condensates emit luminescence which is then passed through an interference loop to obtain an interference fringe pattern. The intensity and phase information of the

petal-shaped condensates can be obtained by solving the interference fringe pattern using quadratic Fourier transform. As shown in FIG. S5, the three petal-shaped exciton-polariton condensates mentioned in the text were measured by feeding them into the interference loop, and the intensity and phase information of each mode were recovered.

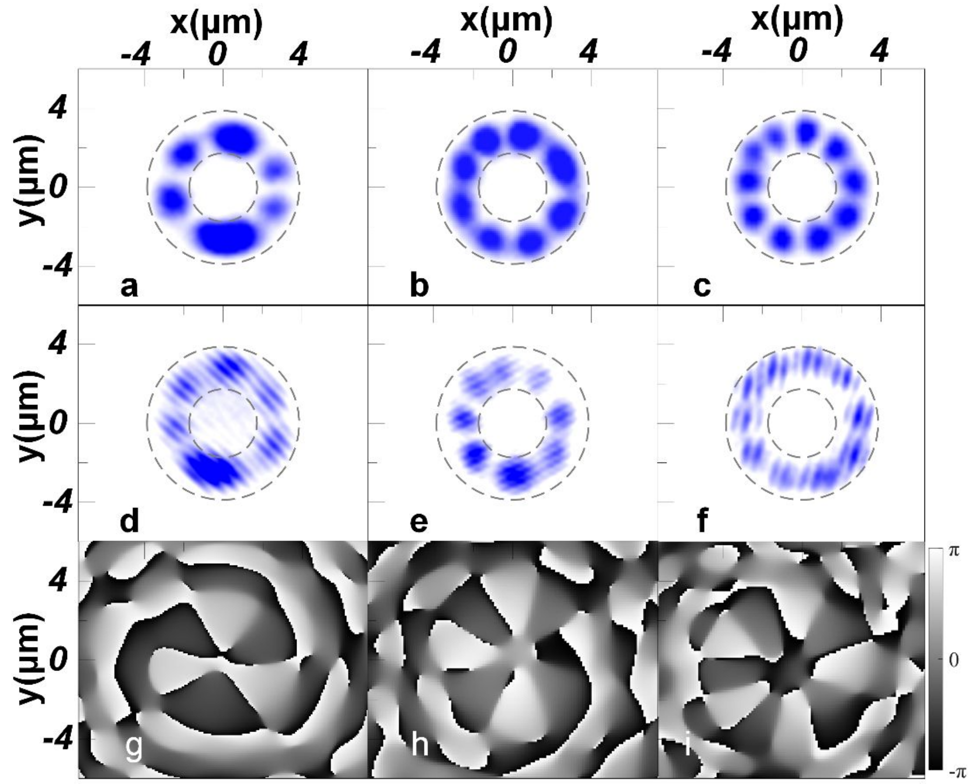

FIG.S5: The first line (a-c) corresponds to the three petal-shaped exciton-polariton condensates observed in the text, and the second line (d-f) shows the measured interference fringe diagram. The dotted gray line indicates the location of the annular well. The phase distribution of the signal light is obtained after solving the interference fringe diagram and is displayed in the third line (g-i).

As shown in Figure S5, the interference fringes (d-f) can be clearly observed through real space interferometry of $l = 3$(a), $l = 4$(b), and $l = 5$(c). The dislocation of fringes can also be observed, indicating the presence of a

phase step at the dislocation. The calculated phase distribution (g-i) compares well with the actual situation.

Supplemental information V. Theoretical model and simulation results

The generation of microcavity exciton-polariton is based on the coupling and excitation of photon and electron hole pair in semiconductor microcavity. For the numerical simulation of microcavity exciton-polariton, the main research object is the superposition state of excited electron-hole pair in semiconductor microcavity and its evolution characteristics. Consider a driven-dissipative Gross-Pitaevskii Equation(GPE) type model[3-5]:

$$i\hbar\frac{\partial}{\partial t}\psi(r,t)=[-\frac{\hbar^2}{2m}\nabla^2-i\hbar\frac{\gamma_c}{2}+g_c|\psi(r,t)|^2+(g_r+i\hbar\frac{R}{2})n(r,t)+V_{ext}(r)]\psi(r,t)$$

$$\frac{\partial n(r,t)}{\partial t}=\left[-\gamma_r-R|\psi(r,t)|^2\right]n(r,t)+P(r,t)$$

$$P(r,t)=P_0\exp\left(-\frac{r^2}{w^2}\right)\exp\left(-\frac{(t-t_0)^2}{w_t^2}\right)$$

In the equations above, the exciton-polariton field is described by the wave function $\psi(r,t)$, where $n(r,t)$ represents the density of the reservoir, and $V_{ext}(r)$ represents the structural barrier that represents the potential of the Bose-Einstein condensate region. The pump light $P(r,t)$ adopts a Gaussian distribution. The loss rate of the condensate and the reservoir are represented

by γ_c and $\gamma_r = 2\gamma_c$, respectively. The repulsive polariton-polariton interaction and resulting nonlinearity in the equation are denoted by g_c. The strength of the polariton-reservoir interaction is given by $g_r = 2g_c$, and the polariton condensation rate is represented by R.

In our optic experiment, the potential well depth of the ring situation well is about 110meV, as measured by the fluorescence spectra of the corresponding perovskites in the annular potential well and no potential well part.

In our simulation calculation, the diameter of the inner and outer rings of the trap is 4-8 μm, and the depth of the potential $V(r,t) = -110meV$.

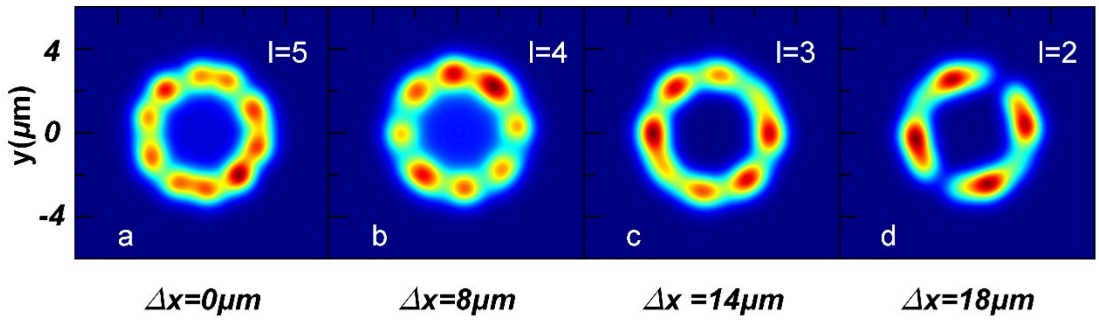

FIG.S6 In the simulation diagram obtained by theoretical calculation, the system evolves from white noise, (a-d) represents the distribution of the final BEC condensate in the system when the center of the Gaussian pump light and the center of the annular microcavity are 0 μm,8 μm,14 μm, and 18 μm respectively.

Through the simulation results, we observed that the rules calculated by the theoretical model are consistent with the phenomena observed in the experiment. It is important to note that during the simulation, we discovered that when the offset is too large, a petal-shaped BEC mode with lower

energy emerges. However, in the actual experiment, the light field distribution at this point becomes highly eccentric, leading to the absence of the $l=2$ mode.

Supplemental information VI. Experimental phenomena of other pump spot sizes

In this study, a Gaussian spot with a 70 μm diameter is employed as the pump light. The impact of the light intensity distribution at the annular well on the distribution of the exciton polariton condensation mode in the annular well is investigated by manipulating the relative position of the spot and the annular well, and a theoretical comparison is made.

In order to study the effect of different light band diameters on the experimental phenomenon, different sizes of Gaussian pump light are utilized along with the same experimental methodology to perform experiments, with the results presented in FIG.S7.

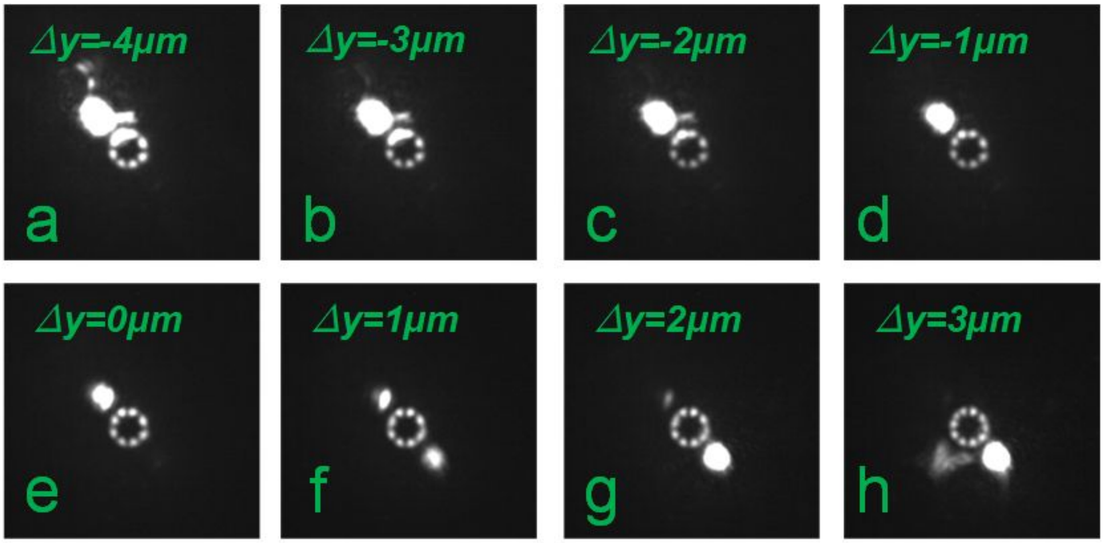

FIG.S7: The phenomenon of exciton polarization mode switching in annular perovskite microcavity when the pump spot diameter is 40 μm. In (e), the relative position of the spot center and the annular well center Δy= 0. In the above figures (a-h), respectively, correspond to the experimental results under different Δy.

Here, we reduce the pump light size to 40 μm and repeat the experiment using the same method as described in the text. The experimental phenomenon is illustrated in the figure above. Compared with the 70 μm spot chosen in the main text, the exciton polariton condensation mode in the annular trap can be achieved by adjusting a small displacement after shrinking the pump light size. Under the pump light parameters of 40 μm, the same three modes as in the text still occur in the same trap. It is noteworthy that due to the smaller spot size, the gradient distribution of the Gaussian pump light at the same size annular trap is more pronounced, and we do not observe a distinct $l = 3$ mode here.

Supplemental information VII. Experimental phenomena in materials of other sizes

In the process of material preparation, we etched various sizes of annular structures on the positive photoresist using photolithography. The experimental results of the annular trap sizes ranging from 4-8 μm are presented in the text, and we explore the influence of the pump light intensity distribution on the emergence of petal-shaped exciton-polariton condensates in the ring. Additionally, we also observed similar phenomena in materials of other sizes.

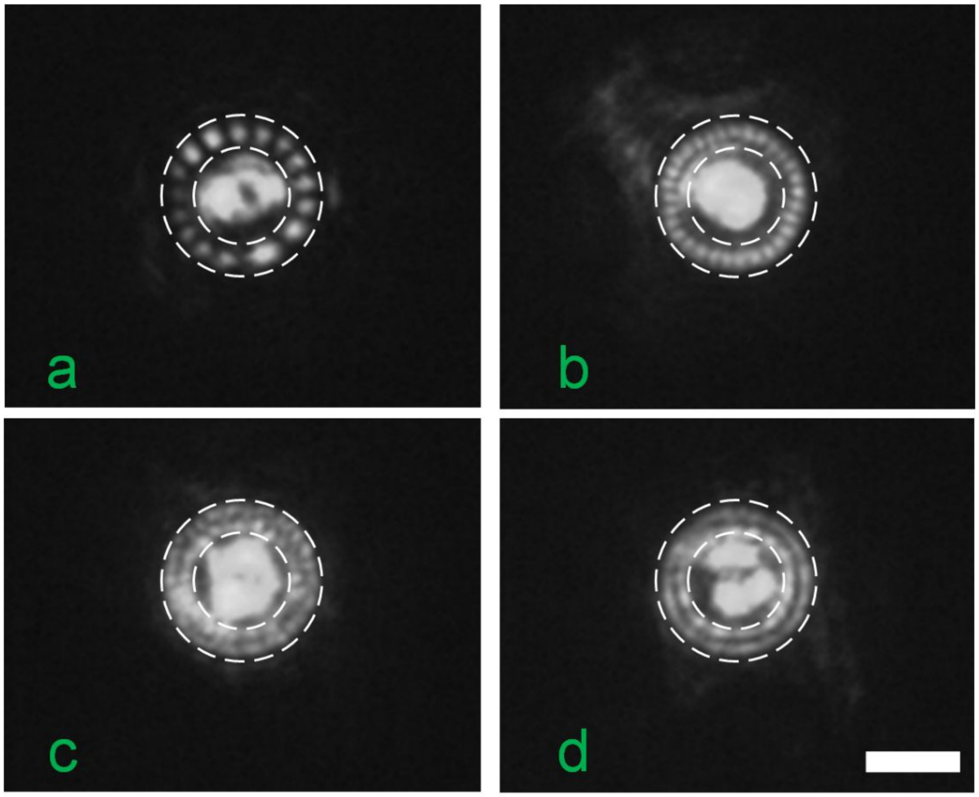

FIG.S8: Experimental results in other annular traps show four patterns observed at this size, which appear to be influenced by changes in the distribution of pump light intensity. The size of the annular traps ranges from 10 to 14 μm. (a) and (b) show observed

petal-shaped exciton-polariton condensates with $l = 8$ and $l = 15$ modes respectively. (c) and (d) not only show the production of petal-shaped exciton-polariton condensates but also the appearance of radial nodes in the radial direction. The scale in the figure represents 10 μm.

As shown in FIG.S8, similar experimental phenomena were also observed in other annular traps prepared by us, with sizes ranging from 4 to 8 μm, as mentioned in the main text. FIG.S8 displays experimental results when the annular microcavity size is between 10 and 14 μm. In particular, FIG.S8 (a) and (b) represent the $l=8$ and $l=15$ modes, respectively. It is worth noting that radial petals are also observed in the annular microcavity of this size, as shown in FIG.S8 (c) and (d). Therefore, petal-shaped exciton-polariton condensates are not only found in the angular direction but also in the radial direction of the annular microcavity.

It is important to note that, due to the large number of petals, some areas of the petals may not be clearly visible during the experimental measurement process. For instance, the clarity of the $l=15$ mode is significantly reduced compared to the $l=8$ mode, especially after the appearance of radial nodes, the observed petals become much blurrier. This can be attributed to the fact that each petal has a fixed phase distribution. In a two-dimensional space with a fixed ring size, each petal is divided into a smaller space on average, making it more unstable.

Furthermore, it can be observed that when the annular microcavity size is between 10 and 14 μm, a Bose-Einstein condensate (BEC) is generated

not only in the annular region but also in the inner circular region surrounded by the annular region. This is because, in the photolithography process, a new system is formed in both the annular and circular regions, which are covered by the perovskite material at the bottom. Therefore, a new BEC system is formed, resulting in luminescence.

Based on this phenomenon, it is hypothesized that the annular microcavity can generate petals similar to a vortex superposition state under the pump light. However, there is a limit to the number of petals that can be "accommodated" within a certain radius of the annular microcavity[6]. If the number of petals exceeds this limit, the system may become unstable and unable to sustain the formation of stable petals. Nevertheless, as the radius of the annular traps increases, the number of petal-shaped exciton-polariton condensates within the annular trap area also increases. Notably, in larger annular microcavities, radial petals may also emerge, resulting in a photoluminescence pattern that more closely resembles the representation of vortex light.

References:

1. Manni, F., et al., *Spontaneous pattern formation in a polariton condensate.* Phys Rev Lett, 2011. **107**(10): p. 106401.
2. Manni, F., et al., *Spontaneous self-ordered states of vortex-antivortex pairs in a polariton condensate.* Physical Review B, 2013. **88**(20).
3. Ma, X. and S. Schumacher, *Vortex Multistability and Bessel Vortices in Polariton Condensates.* Phys Rev Lett, 2018. **121**(22): p. 227404.
4. Ma, X., et al., *Realization of all-optical vortex switching in exciton-polariton condensates.* Nat

Commun, 2020. **11**(1): p. 897.

5. Barkhausen, F., S. Schumacher, and X. Ma, *Multistable circular currents of polariton condensates trapped in ring potentials.* Opt Lett, 2020. **45**(5): p. 1192-1195.
6. Xiong, Z.-Y., et al., *Analysis for vortex superposition state evolution of microcavity exciton polariton excited by ring-shaped pump.* Acta Physica Sinica, 2021. **70**(24).
